# The Bavarian working group on antibiotic resistant pathogens (LARE)

**DOI:** 10.1186/2047-2994-4-S1-P186

**Published:** 2015-06-16

**Authors:** U Kandler, V Lehner-Reindl, G Valenza, C Herr, B Liebl, C Höller

**Affiliations:** 1 Hygiene, Bavarian Health and Food Safety Administration, Erlangen, Germany; 2 Occupational Safety, Bavarian Health and Food Safety Administration, Munich, Germany; 3 Health, Bavarian Health and Food Safety Administration, Oberschleissheim, Germany; 4 Hygiene, Bavarian Health and Food Safety Administration, Oberschleissheim, Germany

## Introduction

Strategies to prevent nosocomial infections and the spreading of multi drug resistant organisms (MDRO) are broadly discussed in the context of hospital care. But MDRO do not only affect hospitals, but also a variety of other health care institutions and medical professions.

## Objectives

The conference of German health ministers recommended in 2006 to form regional networks led by the local public health authorities. The aim of these networks was to address all parties involved in the management of MDRO and to promote the implementation of existing guidelines on prevention and infection control measures.

## Methods

In Bavaria the Ministry of Health initiated in cooperation with the Bavarian Health and Food Safety Authority a two-fold strategy to meet this recommendation. In a first step the Bavarian State Working Group on Antibiotic Resistant Pathogens (LARE) was established in 2008. In a second approach the Bavarian Ministry of Health requested the local public health offices to form networks with all relevant professions and institutions at the regional level.

## Results

Almost all health care associations at the state level participate in this network and strive to implement consistent strategies in the battle against MDRO. Subordinate working groups have successfully prepared and published information for the management of patients with resistant bacteria in the area of occupational health and safety as well as in hospitals and rehabilitation clinics, for patient transportation and in general practice or at home. The annual LARE meetings are well attended by health care workers of all relevant professions. Twice a year the representatives of the participating associations and Bavarian experts get together to discuss actual topics and to consent new publications.

## Conclusion

The established networks at the regional as well as at the state level have led to marked improvements in the communication and collaboration between participating relevant institutions, organisations and various groups of medical professions. However, the challenges for the LARE and the regional networks still stay diverse and will continue in the future.

## Disclosure of interest

None declared.

